# Embryological study of the development of thin membranous dense connective tissues around the esophagus using two fetal cadavers

**DOI:** 10.1007/s10388-026-01198-z

**Published:** 2026-03-25

**Authors:** Yutaka Tokairin, Satoru Muro, kagami Nagai, Kenro Kawada, Masayo Harada, Suthasinee Tharnmanularp, Areeya Jiamjunyasiri, Shikofumi Tei, Yusuke Kinugasa, Keiichi Akita

**Affiliations:** 1 Department of Surgery, Tokyo Metropolitan Toshima Hospital, 33-1 Sakaecho, Itabashi-Ku, Tokyo 173-0015 Japan; 2 Department of Gastrointestinal Surgery, Institute of Science Tokyo, 1-5-45 Yushima, Bunkyo-Ku, Tokyo 113-8510 Japan; 3 Department of Clinical Anatomy, Institute of Science Tokyo, 1-5-45 Yushima, Bunkyo-Ku, Tokyo 113-8510 Japan; 4 Department of Clinical Anatomy, Princess Srisavangavadhana Faculty of Medicine, Chulabhorn Royal Academy, 906 Kamphaeng Phet 6, Talat Bang Khen, Lak Si, Bangkok, 10210 Thailand; 5 Department of Anatomy, Faculty of Medicine, Khon Kaen University, 123 Mitraphap Rd., Nai Muang, Muang, Khon Kaen, 40002 Thailand; 6 Department of Pathology, Tokyo Metropolitan Toshima Hospital, 33-1 Sakaecho, Itabashi-Ku, Tokyo 173-0015 Japan

**Keywords:** Thin membranous dense connective tissues (TMDCT), Histology, Embryology, Mediastinum, Visceral sheath, Vascular sheath, Esophagus

## Abstract

**Objective/Aim:**

We investigated the developmental process of thin membranous dense connective tissue (TMDCT) surrounding the esophagus.

**Methods:**

Horizontal mediastinal sections at 1-mm intervals were prepared from 2 fetal cadavers fixed in 10% formalin at 4 and 8 months of gestation. The sections were stained with hematoxylin and eosin, elastica van Gieson, and Azan. The structures of the dense connective tissues in the cervical and mediastinal regions were compared with those in adult cadavers.

**Results:**

In the superior mediastinum, at 4 months of gestation, homogeneous non-dense fibrous collagenous fibers were observed around the trachea, esophagus, and great vessels. At 8 months of gestation, the difference in collagen fiber density became evident around the great vessels and in parts of the peritracheal and periesophageal regions, forming structures homologous to the vascular and visceral sheaths observed in adults. In the middle-to-lower mediastinum, homogeneous collagen fibers at 4 months of gestation became more organized and denser at 8 months of gestation, especially around the descending aorta and azygos vein. A membranous structure extending bilaterally from the esophagus toward the pulmonary hila was also identified.

**Discussion and conclusion:**

During the mid-to-late fetal development period, regional differentiation of collagen fiber density in the mediastinum becomes apparent, forming the structural basis for the vascular and visceral sheaths in adults. Vascular pulsations and esophageal peristaltic movements are presumed to be the driving forces behind this process.

## Introduction

To rationally perform esophageal cancer surgery, we previously conducted histological investigations of visceral and vascular sheaths [1] originally proposed in macroscopic anatomical studies by Sarrazin et al. [2]. In this study, we found that the dense connective tissue in the upper mediastinum is not cylindrical as described by Sarrazin et al. [2] but rather asymmetrical and that it becomes attenuated and indistinct from the region beneath the aortic arch to the tracheal bifurcation [1]. Furthermore, we demonstrated that, distal to the tracheal bifurcation, dense connective tissue extending beneath the pleura is present between the descending aorta, azygos vein, thoracic duct, and dorsal side of the esophagus, with vascular sheaths similar to those of the upper mediastinum also present around the great vessels [3].

In addition, focusing on the embryological origin of the right subclavian artery and the aortic arch, both derived from the fourth pharyngeal arch artery, we examined serial sections of the connective tissue surrounding the esophagus at the levels cranial and caudal to these arteries, where the recurrent laryngeal nerves recur [4]. We found that the formation of the visceral sheath was indistinct bilaterally at points where the recurrent laryngeal nerves recurred. This phenomenon was presumed to result from the nonparallel and crossing spatial relationship between the esophagus and the arteries derived from the fourth pharyngeal arch, namely the right subclavian artery and aortic arch.

In the present study, the mediastinal connective tissue of a 4-month-old fetus was found to be composed of fibroblasts, collagen fibers, and adipocytes. Collagen fibers form a loose fibrous structure surrounding the major organs. To elucidate how these fetal connective tissue components contribute to the subsequent formation and development of dense connective tissue in adults, histological examinations were performed on an 8-month-old fetal cadaver, and the results were compared with those of adult specimens.

## Methods

Two fetal cadavers at 4 and 8 months of gestation, fixed in 10% formalin and donated to the Department of Clinical Anatomy, Institute of Science Tokyo (Tokyo, Japan), were examined. For comparison with adult specimens, previously reported histological specimens were used. With agreement from the families concerned, the two specimens at 4 and 8 months of gestation had been donated to the Department of Clinical Anatomy, Institute of Science Tokyo, and their use for research was approved by the university ethics committee. The fetuses were obtained by induced or non-induced abortion, and in each case, after the procedure, the obstetrician informed the mother orally about the possibility of fetal donation for research; no attempt to actively encourage donation was made.

A total of six adult cadaveric specimens were included, consisting of three males and three females. The age of the donors ranged from 61 to 101 years, and all specimens were obtained from the same institution. The fixation and staining procedures were performed as described in the cited reference and were identical to those used in the present study.

### Anatomical and histological examinations

Following the method of Muro et al. [1, 5–7], horizontal sections of the fetal mediastinum were prepared from the cervical region to the esophagogastric junction at 1-mm intervals. Paraffin sections of 5-μm thickness were produced and subjected to hematoxylin–eosin (HE), elastica van Gieson (EVG), and Azan staining. The structure of dense connective tissue in the mediastinum was then examined. The gestational age was determined based on the obstetric medical records.

Dense connective tissue was defined as a structure that could be clearly distinguished from the surrounding loose connective tissue on EVG-stained sections, characterized by densely arranged collagen fiber bundles forming a laminar or sheet-like configuration and traceable across serial sections. The evaluation was performed qualitatively.

## Results

The histological findings for each anatomical level are summarized below.

### *Cervicothoracic transitional zone (*Fig. 1b*, arrow a)*

**Fig. 1:**
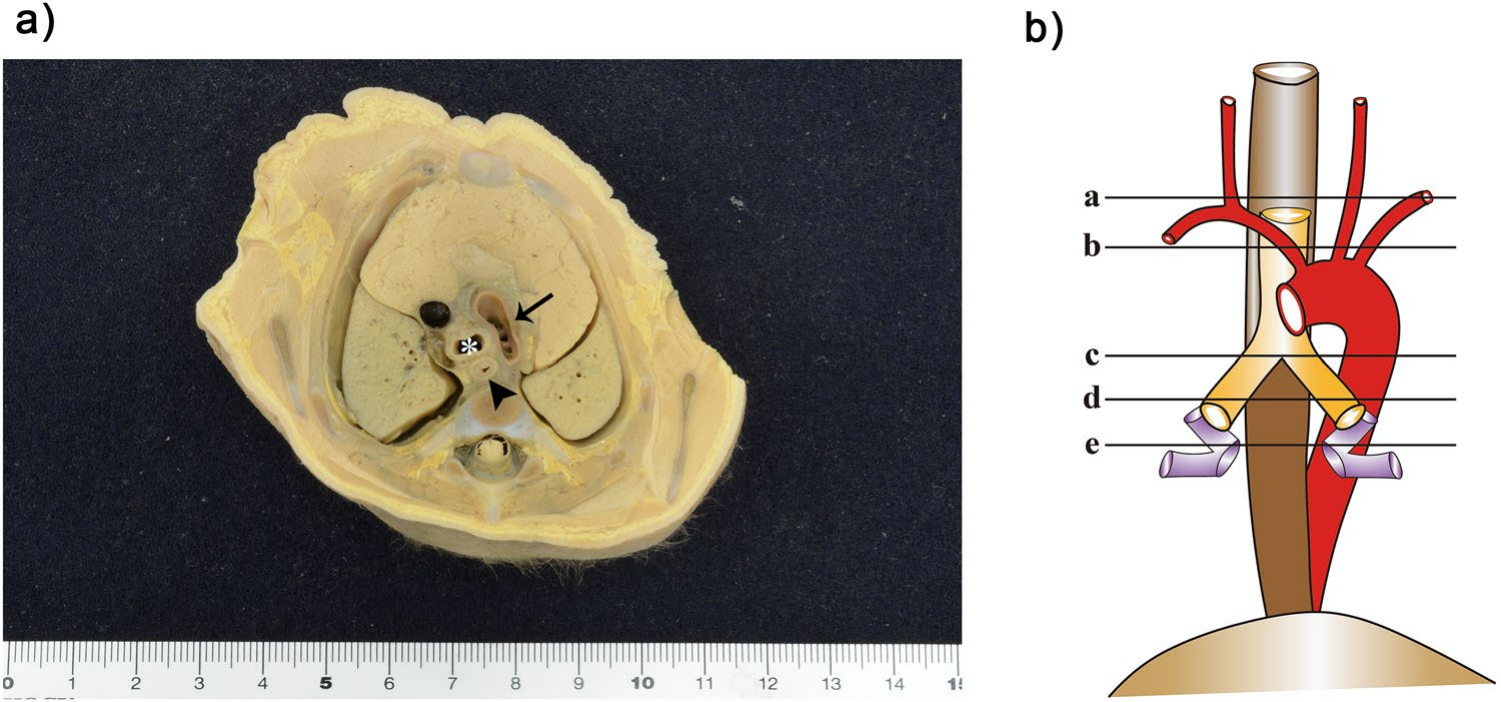
**a** A transverse section including the tissues surrounding the esophagus (arrowhead), trachea (white asterisk), and aortic arch (narrow arrow) was obtained, prepared, and sectioned into 5-μm-thick slices. **b** Transverse sections from cervical–thoracic transitional zone to above the diaphragm, including the vertebra, were prepared and sectioned into 5-μm-thick slices. a: The cervicothoracic transitional zone. b: Between the cervicothoracic transitional zone and the aortic arch. c: Near the bifurcation of the trachea. d: The caudal side of the bifurcation of the trachea. e: At the level of the left atrium

**Fig. 2:**
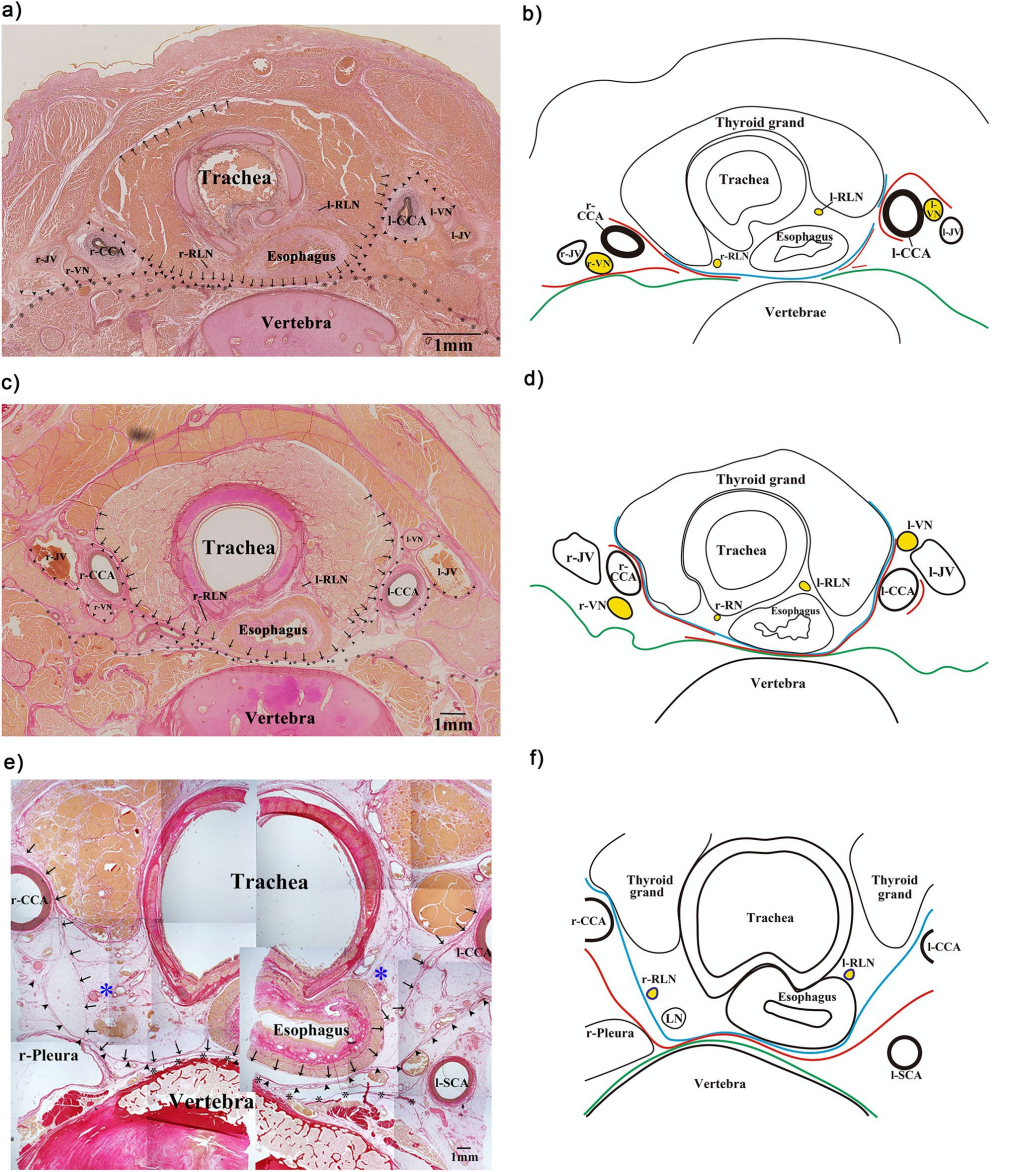
**a** The histological findings in the cervicothoracic transitional zone at 4 months of gestation (EVG staining). A loose collagenous structure was observed surrounding the thyroid gland, trachea, and esophagus. Around the common carotid artery and internal jugular vein, only a homogeneous loose collagen structure was present at 4 months of gestation. The loose collagenous structure considered to be homologous to the adult pretracheal cervical fascia (visceral fascia) (arrow) and the prevertebral cervical fascia (asterisk) were distinguished on both the right and left sides of the cervicothoracic transitional zone. The loose collagenous structure considered to be homologous to the adult alar fascia and carotid sheaths (arrowhead) were observed on both sides. The recurrent laryngeal nerve was observed on both sides. **b** A schematic illustration of **a**. This schematic illustration indicates the structures of the dense connective tissues at cervicothoracic transitional zone at 4 months of gestation. The green line indicates the prevertebral cervical fascia. The red line indicates the loose collagenous structure considered to be homologous to the adult alar fascia and carotid sheaths. The blue line indicates the loose collagenous structure considered to be homologous to the adult pretracheal layer (visceral fascia). The recurrent laryngeal nerves were observed on both sides. **c** The histological findings in the cervicothoracic transitional zone at 8 months of gestation (EVG staining). Distinct differences in the collagen fiber density were noted in the pretracheal tissue at 8 months of gestation, considered to be homologous to the adult pretracheal layer. Around the common carotid artery, the density differences appeared, producing a structure considered to be homologous to the adult carotid sheath. **d** A schematic illustration of **c**. This schematic illustration indicates the structures of the dense connective tissues at cervicothoracic transitional zone at 8 months of gestation. The green line indicates the prevertebral cervical fascia. The red line indicates the loose collagenous structure considered to be homologous to the adult alar fascia and carotid sheaths. The blue line indicates the loose collagenous structure considered to be homologous to the adult pretracheal layer (visceral fascia). The recurrent laryngeal nerves were observed on both sides. **e** The histological findings in the cervicothoracic transitional zone in adult specimens (EVG staining). Around the thyroid gland, trachea, and esophagus, the buccopharyngeal fascia and pretracheal layer were identified, corresponding to the visceral sheath. Around the great vessels in the same zone, the vascular sheath was observed in the corresponding region. Blue asterisks indicate the right and left recurrent laryngeal nerves. **f** A schematic illustration of e, showing the structures of the dense connective tissues at the cervicothoracic transitional zone in adult specimens. The green line indicates the prevertebral cervical fascia. The red line indicates the alar fascia and carotid sheaths. The blue line indicates the pretracheal layer (visceral fascia). The recurrent laryngeal nerves were identified bilaterally. Panels e and f are modified from Tokairin et al., Ref. 1, with permission. *l-CCA* left common carotid artery, *l-JV* left jugular vein, *l-RLN* left recurrent laryngeal nerve, *l-VN* left vagus nerve, *r-CCA* right common carotid artery, *r-JV* right jugular vein, *r-RLN* right recurrent laryngeal nerve, *r-VN* right vagus nerve

Around the thyroid gland, trachea, and esophagus, a loose fibrous structure composed of relatively uniform and loosely arranged collagen fibers was present at 4 months of gestation (Fig. 2a, b). However, this layer was not very clearly demarcated. At 8 months of gestation, differences in collagen fiber density became evident in the same regions (Fig. 2c, d). These areas were homologous to the pretracheal layer observed in adults. In adults, the buccopharyngeal fascia and pretracheal layer were identified in the corresponding region (Fig. 2e, f), representing the mature configuration corresponding to the visceral sheath.

Around the great vessels in the same zone, only a uniform loose fibrous structure was observed at 4 months of gestation (Fig. 2a, b), whereas at 8 months of gestation, differences in fiber density appeared (Fig. 2c, d), in a region homologous to that of the vascular sheath in adults. The vascular sheath was observed in the corresponding region in adult specimens, consistent with the mature configuration (Fig. 2e, f).

### *Between the cervicothoracic transitional zone and the aortic arch (cranial end of the mediastinum, *Fig. 1b*, arrow b)*

**Fig. 3:**
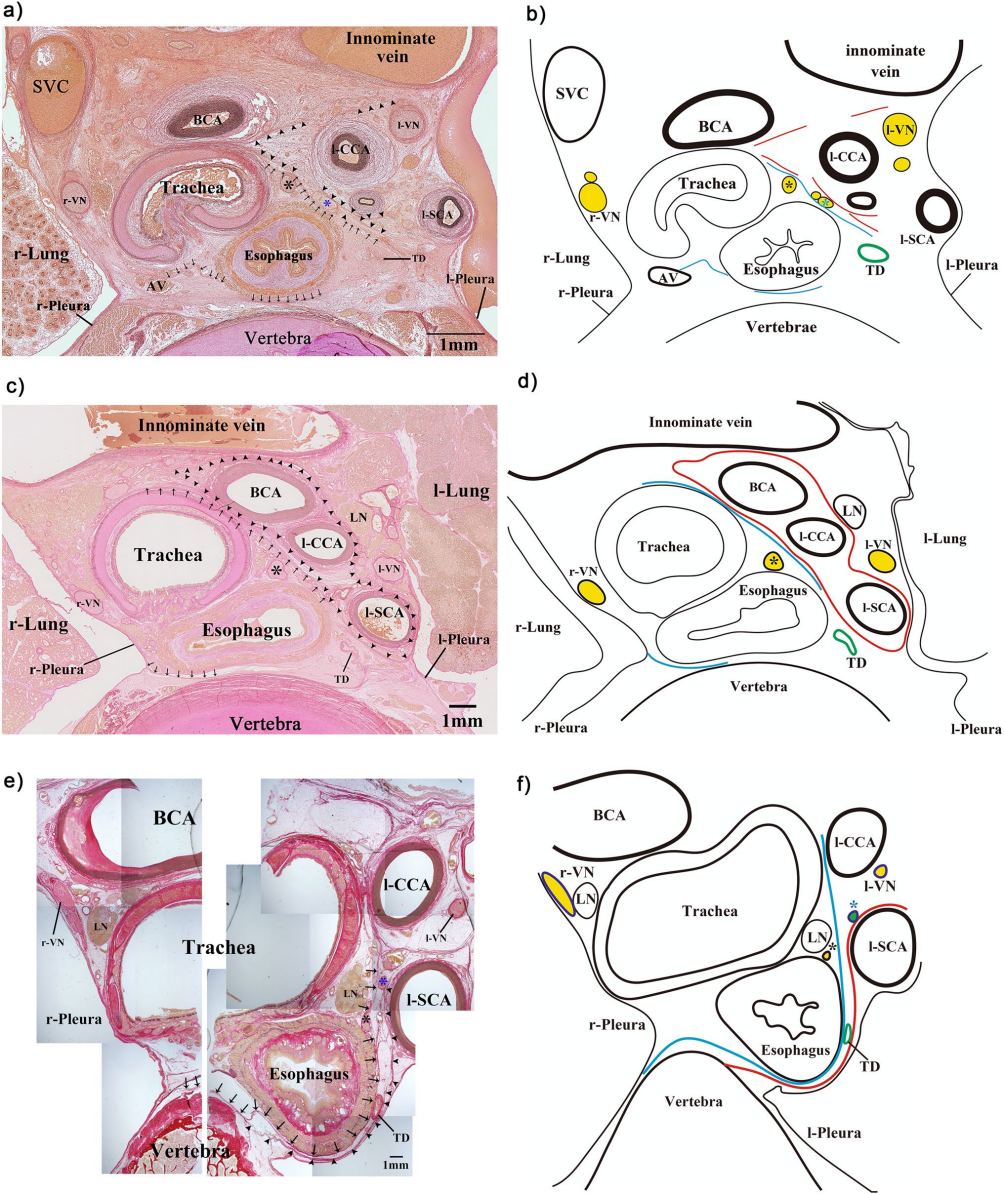
**a** The histological findings between the cervicothoracic transitional zone and the aortic arch at 4 months of gestation (EVG staining). A relatively homogeneous loose collagenous structure was present around the brachiocephalic artery, left common carotid artery, and left subclavian artery, with only slight density variations at the margins. The ramus cardiacus of the sympathetic nerve (blue asterisk) is observed on this structure. Only a loose fibrous structure was observed around the trachea and esophagus. The left recurrent laryngeal nerve (black asterisk) was identified on the visceral side of this membranous structure. Around the thoracic duct, a homogeneous collagen structure was slightly present, but the boundary between the esophagus and the thoracic duct was indistinct. **b** A schematic illustration of **a**. The thin membranous structure considered to be homologous to the adult visceral sheath is indicated with a blue line, and the structure considered to be homologous to the adult vascular sheath is indicated with a red line. The development of these sheaths was still incomplete, and the later structure was more prominently developed than the other. **c** The histological findings between the cervicothoracic transitional zone and the aortic arch at 8 months of gestation (EVG staining). Collagen density differences became pronounced, forming a structure considered to be homologous to the adult vascular sheath. A distinct difference in collagen fiber density had become apparent on the left side of the trachea and esophagus, forming a structure considered to be homologous to the adult visceral sheath. In addition, the dense connective tissue was identified on the dorsal side of the esophagus, extending toward the right pleura. Furthermore, collagen density differences became apparent, with the thoracic duct situated between the structure considered to be homologous to the adult vascular sheath and the dorsal side of the esophagus. **d** A schematic illustration of **c**. The thin membranous structure considered to be homologous to the adult visceral sheath is indicated with a blue line, and the structure considered to be homologous to the adult vascular sheath is indicated with a red line. The development of the structure considered to be homologous to the adult visceral sheath was still incomplete; however, the structure considered to be homologous to the adult vascular sheath was considerably more advanced. **e** The histological findings between the cervicothoracic transitional zone and the aortic arch in adult specimen (EVG staining). A more clearly delineated thin membranous dense connective tissue structure under the right pleura (arrow) ran behind the esophageal wall and between the thoracic duct and the esophagus, corresponding to the visceral sheath. The left recurrent laryngeal nerve (black asterisk) and adjacent lymph nodes were located on the organ side of this structure. Another well-defined thin membranous dense connective tissue structure (black arrowhead) extended from the left side of the vertebral region toward the ventral side of the left pleura and around the left subclavian artery, corresponding to the vascular sheath. The ramus cardiacus of the sympathetic nerve (blue asterisk) was observed on this structure. **f** A schematic illustration of e. The blue line indicates the visceral sheath. The red line indicates the vascular sheath. The left recurrent laryngeal nerve (black asterisk) and adjacent lymph nodes are located on the organ side of the visceral sheath. Panels e and f are modified from Tokairin et al., Ref. 1, with permission. *BCA* brachiocephalic artery, *l-CCA* left common carotid artery, *l-Lung* left lung, *LN* lymph node, *l-Pleura* left pleura, *l-SCA* left subclavian artery, *l-VN* left vagal nerve, *r-Lung* right lung, *r-Pleura* right pleura, *r-VN* right vagal nerve, *TD* thoracic duct, *black asterisk:* left recurrent laryngeal nerve, *blue asterisk:* ramus cardiacus of the sympathetic nerve

Around the brachiocephalic, left common carotid, and left subclavian arteries, a relatively uniform loose collagenous structure was present at 4 months of gestation, with only minimal density differences at the periphery (Fig. 3a, b). At 8 months of gestation, differences in collagen fiber density were evident in the same regions (Fig. 3c, d). These regions were homologous to the vascular sheath observed in adults. A more clearly delineated thin membranous structure composed of dense connective tissue was identified in the corresponding region in adult specimens, representing the vascular sheath (Fig. 3e, f).

Around the trachea and esophagus, only a loose fibrous structure was observed at 4 months of gestation (Fig. 3a, b). At 8 months of gestation, a distinct difference in collagen fiber density was apparent on the left side of the trachea and esophagus (Fig. 3c, d), forming a structure homologous to the visceral sheath observed in adults. In addition, dense connective tissue was identified on the dorsal side of the esophagus, extending toward the right pleura. In adult specimens, a more clearly defined thin membranous structure composed of dense connective tissue enclosed the esophagus, the trachea, and adjacent lymphatic structures on the left side, corresponding to the visceral sheath (Fig. 3e, f).

Around the thoracic duct, only a poorly demarcated loose collagenous structure was observed at 4 months of gestation (Fig. 3a, b). At 8 months of gestation, a fibrous structure with distinct density differences was formed (Fig. 3c, d), forming a structure homologous to the vascular sheath observed in adults. The thoracic duct was located between this perivascular fibrous structure and the dorsal side of the esophagus. In adult specimens, the thoracic duct was separated from the esophagus by the visceral sheath and was located between the visceral and vascular sheaths (Fig. 3e, f).

### *Near the bifurcation of the trachea zone (*Fig. 1b*, arrow c)*

**Fig. 4:**
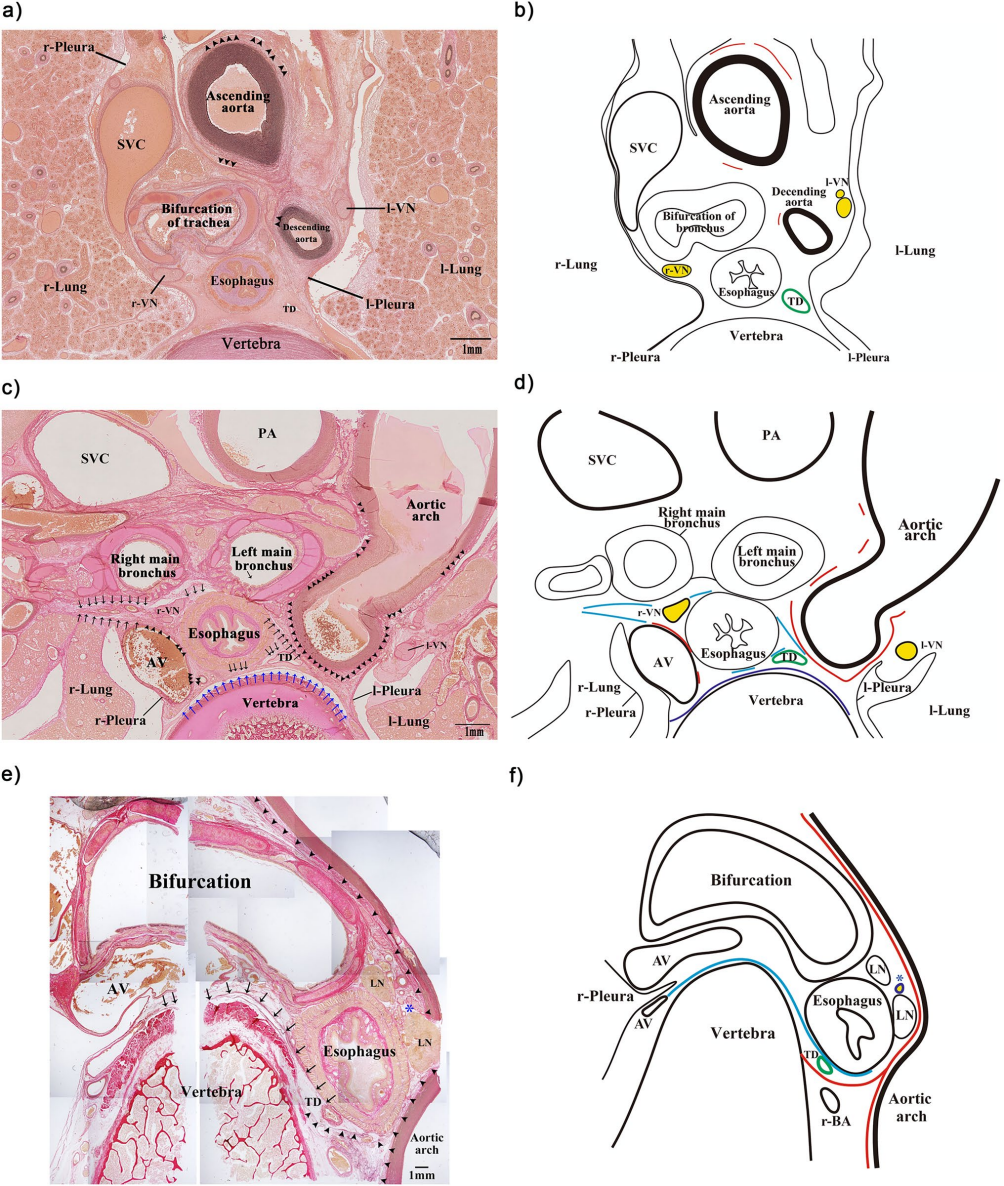
**a** The histological findings near the bifurcation of the trachea at 4 months of gestation (EVG staining). Only a homogeneous and loose collagenous structure was observed around the esophagus, without evident density differences. Around the aortic arch and azygos arch, a loose collagenous structure was homogenously distributed. In addition, a homogeneous and loosely arranged collagenous structure was observed between the thoracic duct and the esophagus; however, no distinct dense connective tissue separating the two structures could be identified. **b** A schematic illustration of **a**. This schematic illustration shows the structures near the bifurcation of the trachea at four months of gestation. The structure considered to be homologous to adult vascular sheath is indicated with a red line. The structure considered to be homologous to the adult visceral sheath was scarcely identifiable, and only a slight development of the structure considered to be homologous to the adult vascular sheath was observed. **c** The histological findings near the bifurcation of the trachea at 8 months of gestation (EVG staining). Membranous collagen structures extended bilaterally from the esophagus: on the right, to the right pulmonary hilum which contains arteries and the vagus nerve; on the left, to the left subpleural region. Distinct density variations were observed at the anterior and the posterior to these membranous structures, forming a structure considered to be homologous to the adult visceral sheath. Around the aortic arch and azygos arch, density differences were distinct, yielding the structure considered to be homologous to adult vascular sheath. In addition, the thoracic duct was located dorsal to the structure considered to be homologous to the adult visceral sheath on the dorsal side of the esophagus. **d** A schematic illustration of **c**. This schematic illustration shows the structures near the bifurcation of the trachea at 8 months of gestation. The thin membranous structure considered to be homologous to the adult visceral sheath is indicated with a blue line, and the structure considered to be the homologous to the adult vascular sheath is indicated with a red line. The structure considered to be homologous to the adult visceral sheath was partially identifiable, and only partial development of the structure considered to homologous to adult vascular sheath was observed around the aortic arch and azygos arch. **e** The histological findings near the bifurcation of the trachea in adult specimens (EVG staining). A well-defined thin membranous dense connective tissue structure under the right pleura (arrow) ran behind the esophageal wall and between the thoracic duct and the esophagus, corresponding to the visceral sheath. This structure became indistinct on the left side of the esophagus. Another thin membranous dense connective tissue structure (arrowhead) was observed around the aortic arch and the dorsal side of the thoracic duct, corresponding to the vascular sheath. **f** A schematic illustration of e. The blue line indicates the visceral sheath. The red line indicates the vascular sheath. Panels e and f are modified from Tokairin et al., Ref. 1, with permission. *AV* azygos vein, *l-Lung* left lung, *LN* lymph node, *l-PA* left pulmonary artery, *l-Pleura* left pleura, *l-VN* left vagus nerve, *r-PA* right pulmonary artery, *r-Pleura* right pleura, *r-VN* right vagus nerve, *SVC* superior vena cava, *TD* thoracic duct, *blue asterisk*: left recurrent laryngeal nerve

Around the esophagus, a homogeneous and loose collagenous structure was observed at 4 months of gestation, with no apparent differences in collagen fiber density (Fig. 4a, b). At 8 months of gestation, a collagenous structure formed bilaterally along the esophagus, extending toward the right pulmonary hilum on the right side and to the left subpleural region on the left side (Fig. 4c, d). Because this structure formed a continuous plane associated with small vessels and adjacent loose connective tissue, it resembled a mesentery and is hereafter referred to as “mesentery-like.” Distinct differences in collagen fiber density were observed on the ventral and dorsal sides of the mesenteric structure, forming a structure homologous to the visceral sheath observed in adults, representing progressive development of the sheath from 4 to 8 months of gestation and into adulthood (Fig. 4e, f).

Around the aortic and azygos arches, a loose collagenous structure was homogeneously distributed at 4 months of gestation (Fig. 4a, b), whereas at 8 months of gestation, distinct density differences were observed (Fig. 4c, d). These regions were homologous to the vascular sheath observed in adults. A thin membranous dense connective tissue structure was identified around the aortic arch in adult specimens, consistent with further development of the vascular sheath (Fig. 4e, f).

Around the thoracic duct, the dense connective tissue separating it from the esophagus was indistinct at 4 months of gestation (Fig. 4a, b), whereas clear differences in fiber density were evident at 8 months of gestation (Fig. 4c, d). This region corresponds to the visceral sheath in adults. In adult specimens, the thoracic duct was separated from the esophagus by the visceral sheath and was located between the visceral and vascular sheaths, reflecting the developmental progression of these sheath structures (Fig. 4e, f).

### *The caudal side of the bifurcation of the trachea (*Fig. 1b*, arrow d)*

**Fig. 5:**
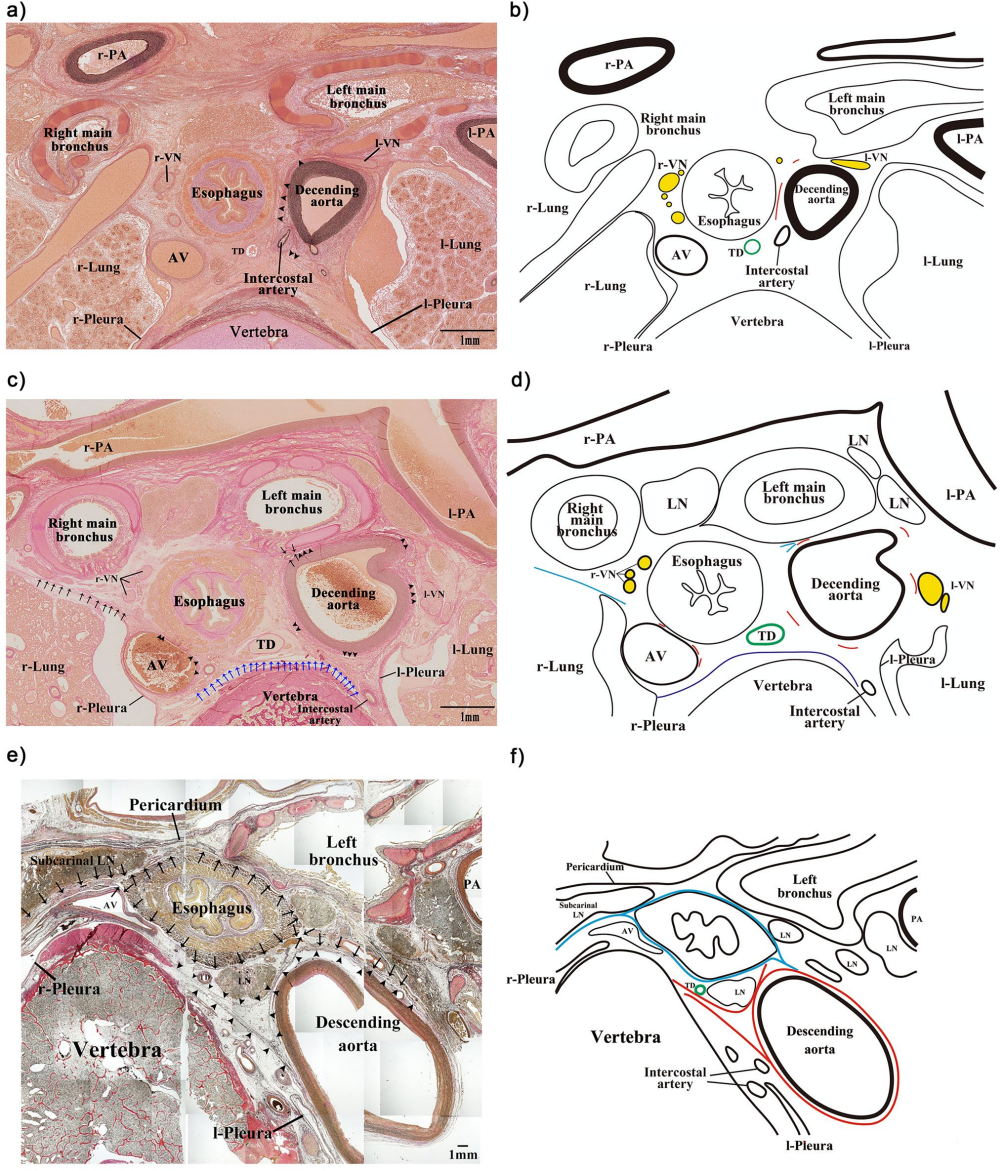
**a** The histological findings of the caudal side of the bifurcation of the trachea at 4 months of gestation (EVG staining). A homogeneous and loosely arranged collagenous structure was distributed around the esophagus, particularly extending bilaterally toward the pulmonary hila. The structures around the descending aorta, the azygos vein, and the thoracic duct exhibited a developmental pattern similar to that at the tracheal bifurcation level. **b** A schematic illustration of **a**. The thin membranous structure considered to be homologous to the adult vascular sheath is indicated by the red line. The development of both visceral and vascular sheaths is rarely observed. **c** Histological findings of the caudal side of the bifurcation of the trachea at 8 months of gestation (EVG staining). The collagenous structure connecting to the pulmonary hila had become thinner; on the right side, it extended laterally toward the right pulmonary hilum, whereas on the left side, it passed ventral to the descending aorta and continued toward the left pulmonary hilum. The development of the homologous to the adult visceral sheath (black arrow) was slightly more evident than that at 4 months of gestation. Distinct differences in collagen fiber density were identified on the dorsal side of the thoracic duct (blue arrow), extending toward the right pleura. **d** A schematic illustration of **c**. This schematic illustrates the structures of the caudal side of the bifurcation of the trachea at 8 months of gestation. The thin membranous structure considered to be homologous to the adult visceral sheath is indicated by the blue line, and the structure considered to be homologous to the adult vascular sheath is indicated by the red line. The development of the homologous to the adult visceral sheath was slightly more evident than that at 4 months of gestation. Only partial development of the structure homologous to the adult vascular sheath was observed around the descending aorta and azygos vein. **e** The histological findings of the caudal side of the bifurcation of the trachea in adult specimens (EVG staining). A well-defined thin membranous dense connective tissue structure surrounding the esophagus (arrow) was observed on the dorsal side, corresponding to the posterior part of the visceral sheath. Another thin membranous dense connective tissue structure (arrowhead) extended from the dorsal side of the thoracic duct and integrated with the adventitia of the descending aorta, corresponding to the vascular sheath. The thoracic duct and adjacent lymph nodes were located between these structures. A thin membranous structure was also observed on the ventral side of the esophagus, corresponding to the anterior part of the visceral sheath. **f** A schematic illustration of e. The blue line indicates the visceral sheath. The red line indicates the vascular sheath. Panels e and f are modified from Tokairin et al., Ref. 3, with permission. *AV* azygos vein, *l-Lung* left lung, *LN* lymph node, *l-PA* left pulmonary artery, *l-Pleura* left pleura, *l-VN* left vagus nerve, *PA* pulmonary artery, *r-Lung* right lung, *r-PA* right pulmonary artery, *r-Pleura* right pleura, *r-VN* right vagus nerve, *TD* thoracic duct

Around the esophagus, only a homogeneous and loosely arranged collagenous structure was distributed at 4 months of gestation, with little variation in fiber density (Fig. 5a, b). At 8 months of gestation, mesentery-like collagenous structures formed bilaterally along the esophagus, extending to the right pulmonary hilum on the right side and to the left pulmonary hilum via the ventral side of the descending aorta on the left side (Fig. 5c, d). The locations of these collagenous structures were homologous to the visceral sheath observed in adults. Thin membranous dense connective tissue structures were identified along the dorsal and ventral sides of the esophagus in adult specimens, corresponding to the visceral sheath (Fig. 5e, f).

Around the descending aorta, azygos vein, and thoracic duct, a developmental pattern similar to that observed at the tracheal bifurcation level was observed (Fig. 5a–d). A thin membranous dense connective tissue structure corresponding to the vascular sheath extended toward and integrated with the adventitia of the descending aorta, with the thoracic duct and adjacent lymph nodes located between this structure and the visceral sheath in adult specimens (Fig. 5e, f).

These findings indicate that the visceral and vascular sheath structures progressively develop from 4 to 8 months of gestation and ultimately form structures homologous to the visceral and vascular sheaths observed in adults.

### *At the level of the left atrium (cranial portion of the lower mediastinum, *Fig. 1b*, arrow e)*

**Fig. 6:**
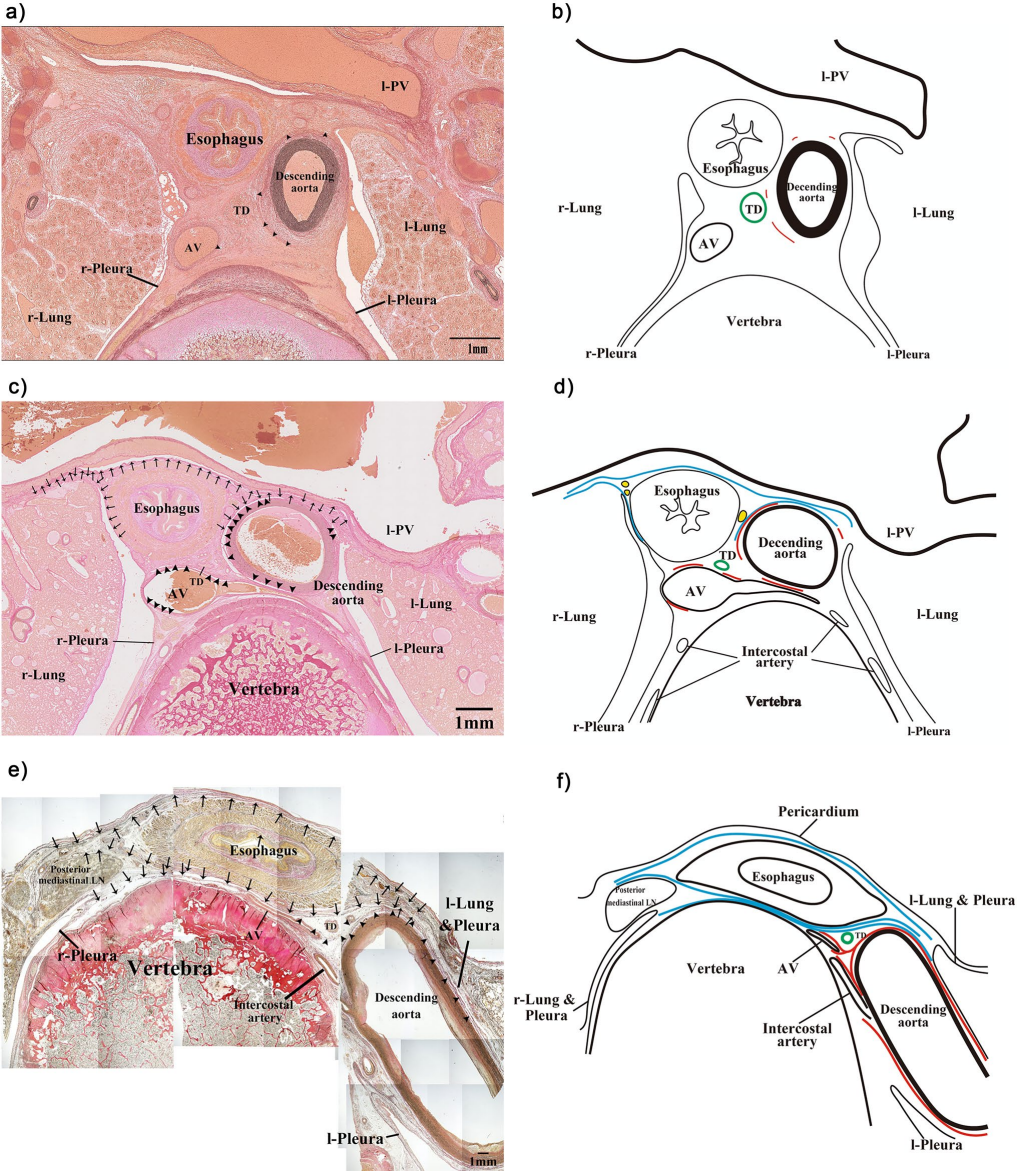
**a** Histological findings at the level of the left atrium at 4 months of gestation (EVG staining). A homogeneous and loosely arranged collagenous structure was distributed around the esophagus, particularly extending bilaterally toward the pulmonary hila. **b** A schematic illustration of **a**. The thin membranous structure, considered to be homologous to the adult vascular sheath, is indicated by a red line. The development of the homologous to the adult visceral sheath was scarcely observed. **c** Histological findings at the left atrium at 8 months of gestation (EVG staining). The collagenous structure connecting to the pulmonary hila had become thinner; on the right side, it extended laterally toward the right pulmonary hilum, whereas on the left side, it passed ventral to the descending aorta and continued toward the left pulmonary hilum. Differences in collagen fiber density were observed within the bilateral mesenteric-like structures, resulting in a configuration homologous to the adult visceral sheath (arrow). The differences in the structures surrounding the descending aorta, azygos vein and the thoracic duct (arrowhead) were slightly more evident than that at four months of gestation. **d** A schematic illustration of **c**. This schematic illustrates the structures at the level of the left atrium at 8 months of gestation. The thin membranous structure considered to be homologous to the adult visceral sheath is indicated by the blue line, and the structure considered to be homologous to the adult vascular sheath is indicated by the red line. The developments of the homologous to the adult visceral sheath and vascular sheath were slightly more evident than that at four months of gestation. **e** Histological findings at the level of the left atrium in adult specimens (EVG staining). A well-defined thin membranous dense connective tissue structure between the esophagus and the thoracic duct was identified, corresponding to the posterior part of the visceral sheath (arrow). Part of this structure extended toward the subpleural region and was continuous with a thin membranous structure on the ventral side of the esophagus, corresponding to the anterior part of the visceral sheath (arrow). Another thin membranous dense connective tissue structure was observed around the azygos vein and extended toward the dorsal side of the thoracic duct to reach the adventitia of the descending aorta, corresponding to the vascular sheath (arrowhead). **f** A schematic illustration of e. The blue line indicates the visceral sheath. The red line indicates the vascular sheath. Panels e and f are modified from Tokairin et al., Ref. 3, with permission. *AV* azygos vein, *l-Lung* left lung, *LN* lymph node, *l-Pleura* left pleura, *l-PV* left pulmonary vein, *l-VN* left vagus nerve, *r-Lung* right lung, *r-Pleura* right pleura, *r-VN* right vagus nerve, *TD* thoracic duct

Around the esophagus, a homogeneous and loosely arranged collagenous structure extended bilaterally toward the pulmonary hila at 4 months of gestation, with no marked differences in collagen fiber density (Fig. 6a, b). At 8 months of gestation, these collagenous structures became thinner, extending to the right pulmonary hilum on the right side and to the left pulmonary hilum via the ventral side of the descending aorta on the left side (Fig. 6c, d). Distinct differences in collagen fiber density developed within these mesentery-like structures, forming a structure homologous to the visceral sheath observed in adults. A more clearly delineated thin membranous dense connective tissue structure corresponding to the visceral sheath was identified along both the ventral and dorsal sides of the esophagus in adult specimens (Fig. 6e, f).

The differences in the structures around the descending aorta, azygos vein, and thoracic duct at 4 and 8 months of gestation were similar to those observed at the level of the tracheal bifurcation (Fig. 6a–d). In adult specimens, a well-defined thin membranous dense connective tissue structure was observed around the azygos vein, extending toward the dorsal side of the thoracic duct and integrating with the adventitia of the descending aorta, corresponding to the vascular sheath (Fig. 6e, f).

## Discussion

We previously reported dense connective tissue surrounding the esophagus of an adult. Regarding the formation of connective tissue (sheath or fascia) within the mediastinum, although macroscopic anatomical studies on the Morozow’s ligament in the middle and lower mediastinum [8] and magnetic resonance imaging (MRI)-based reports [9] have been published, no studies employing histological methods have been documented. In the present study, to elucidate how these connective tissue structures develop, including those in the superior mediastinum, we examined the differences in the dense connective tissue surrounding the esophagus at representative levels from the cervical to the lower mediastinum in cadavers at 4 and 8 months of gestation and compared them with those in adults.

The principal findings are as follows:

1. Upper mediastinum: Development of collagenous structures around the blood vessels was observed as early as 4 months of gestation. In contrast, around the trachea and esophagus, a collagenous structure was noted on the left side from 4 months of gestation, and at 8 months of gestation, distinct differences in the collagen fiber density had developed on the dorsal side of the esophagus. However, on the right side, the collagenous structure remained poorly developed. These findings represent the developmental process of visceral sheath formation.
2. Middle and lower mediastinum: At 4 months of gestation, in the middle and lower mediastinum, the esophagus was connected to both pulmonary hila through a collagenous layer. At 8 months of gestation, distinct differences in the collagen fiber density were observed on the ventral and dorsal sides of the collagenous structures, extending from the esophagus toward the pulmonary hila. A slightly denser region of collagen fibers was observed on the ventral side of the esophagus, suggesting that this finding represents the early stage of visceral sheath development observed in the middle and lower mediastinum of adults.

From an embryological perspective, the dense connective tissue that separates the esophagus from the thoracic duct and the descending aorta in the middle and lower mediastinum of adults, namely the visceral sheath, does not appear to be a structure formed according to a predetermined developmental plan but rather one that may have developed secondarily due to certain postnatal factors. Specifically, three possibilities may be considered: (1) dense connective tissue may have formed around the esophagus as a result of esophageal peristaltic movements; (2) after the fusion of the bilateral pleurae, residual subpleural dense connective tissue may have contributed to its formation; and (3) even without pleural fusion, dense connective tissue may have developed beneath the subpleural region. In the present fetal specimens, no well-defined fascial structures were identified in the mediastinum, such as Toldt’s fascia in the abdomen [10] or Denonvilliers’ fascia in the pelvic region [11]. In our previous study on adult specimens, we reported that the posterior layer of the visceral sheath corresponds to Morozow’s ligament [3]. Based on the present embryological findings, we further inferred that the Morozow ligament represents dense subpleural connective tissue, namely the dorsal component of the visceral sheath.

In the present study, the interpleural distance was consistently wide on both sides at 4 and 8 months of gestation, and in adults. Considering that differences in collagen fiber density were already formed dorsal to the esophagus from 8 months of gestation, possibilities (1) and (3) appear to be more plausible. However, the possibility that some mechanism (2) occurred immediately after birth, associated with the rapid expansion of the lungs, cannot be ruled out. Therefore, further investigation is required.

It is well recognized that physical stimuli are involved in promoting collagen fiber proliferation, which may explain the development of differences in connective tissue density during fetal growth [12, 13].

The formation of the visceral sheath in the cervical region is reportedly influenced by arterial pulsation, peristaltic movement, swallowing, and respiration [14, 15]. Because the visceral sheath is continuous with the mediastinal region, it is possible that similar mechanical stimuli may influence collagen fiber proliferation, which may be associated with the formation of structures, such as vascular and visceral sheaths in the mediastinum. Given that no distinct dense connective tissue demarcating the boundary between the esophagus and thoracic duct was observed even at 8 months of gestation, it is conceivable that peristaltic movements associated with swallowing after birth might play a role in the formation of the visceral sheath in addition to arterial pulsation.

The superior mediastinum may represent a region compatible with the hypothesis that vascular pulsation contributes to the development of dense connective tissue structures. In this region, the dense connective tissue on the left side of the esophagus appears to be well developed from the early fetal stage, whereas that on the right side remains less distinct. At 4 months of gestation, the arteries generally followed the same course as in adults: the brachiocephalic artery was located ventral to the trachea, the left common carotid artery and the left subclavian artery were situated on the left side of the trachea, and the esophagus was positioned slightly to the left and dorsal to the trachea. On the left side of the esophagus, the great vessels were in contact with each other through a homogeneous and loosely arranged fibrous structure. In contrast, the brachiocephalic artery, which is a great vessel on the right side, was located on the left ventral side of the trachea and not in direct contact with the esophagus. The right wall of the esophagus was separated from the right pleura by a relatively wide homogeneous and loose fibrous structure with no intervening major structures. Although differences in collagen fiber density appeared early on the left side of the esophagus, these differences were indistinct on the right side. This finding may suggest that arterial pulsation may contribute to the formation of dense connective tissue around the esophagus. Considering the left–right asymmetry of the great vessels, this mechanism might also be related to the asymmetrical development of the sheath observed in the superior mediastinum.

On the dorsal side of the esophagus, differences in collagen fiber density were observed as early as 4 months of gestation. Embryologically, the trachea originates from the esophagus, and in certain regions, the membranous portions of the trachea and esophagus exhibit partial fusion. Therefore, it is conceivable that during esophageal peristalsis, the side facing the membranous portion of the trachea may move less freely, whereas the dorsal portion of the esophagus may exhibit greater mobility. This differential mobility could lead to variations in collagen fiber density on the dorsal side of the esophagus, which, in turn, may be associated with the subsequent formation of the visceral sheath. However, these interpretations remain speculative and require further investigation. If investigations across additional developmental stages (e.g., infancy, early childhood, later childhood, and young adulthood) become feasible, these observations may be advanced from a hypothesis toward evidence.

From a clinical standpoint, the present developmental findings may have implications for the management of the thoracic duct and surrounding lymphatic nodes during esophagectomy. Fetal observations suggest that the thoracic duct and adjacent lymphatic structures are organized within a compartment defined by the developing perivascular and periesophageal connective tissue. In adults, this sheath-based structure may underlie the anatomical separation between the thoracic duct and adjacent structures.

This perspective may be applicable when deciding whether to preserve or dissect the thoracic duct and its adjacent lymph nodes. Thus, the developing structure demonstrated in this study may provide a structural basis for surgical strategies involving the thoracic duct and its associated lymph nodes.

Several limitations associated with the present study warrant mention. First, only two fetal cadavers, one at 4 months of gestation and one at 8 months of gestation, were examined, which represents a small sample size. Second, the present histological observations differ methodologically from macroscopic anatomical studies, such as those by Morozow and from the exquisite structural findings observed in surgical practice; therefore, direct comparisons or definitive conclusions regarding their similarities or differences are difficult to make. In addition, the evaluation of collagen fiber density was based on morphological assessment using EVG staining, including staining intensity, laminar configuration, and regional differences, rather than on quantitative image analysis. However, the aim of this study was to identify the presence and spatial organization of collagen-dense connective tissue structures and their developmental continuity, rather than to quantify collagen density. The consistent laminar structure observed across serial sections and anatomical levels supports the reproducibility of the findings, although further quantitative studies are warranted.

However, while the number of specimens examined was limited, the findings are highly suggestive. The present embryological and histological analyses are likely to reflect the actual tissue architecture encountered in clinical practice, providing valuable insights for performing precise surgical procedures along the dense connective tissue structures surrounding the esophagus.

## Conclusion

During the fetal period, the formation of the vascular and visceral sheaths was confirmed to occur sequentially. These processes are presumed to be driven primarily by pulsation of the great vessels and peristaltic movements of the esophagus.

## Data Availability

The data that support the findings of this study are not openly available for reasons of sensitivity; however, they are available from the corresponding author upon reasonable request.
